# Proteomics approaches to fibrotic disorders

**DOI:** 10.1186/1755-1536-5-S1-S10

**Published:** 2012-06-06

**Authors:** Marjan Gucek

**Affiliations:** 1 NHLBI Proteomics Core , National Heart, Lung, and Blood Institute (NHLBI), National Institutes of Health, 10 Center Drive, Bethesda, MD 20892, USA

## Abstract

This review provides an introduction to mass spectrometry based proteomics and discusses several proteomics approaches that are relevant in understanding the pathophysiology of fibrotic disorders and the approaches that are frequently used in biomarker discovery.

## Introduction

What are the changes in protein levels that occur during or because of fibrosis? What are the signaling mechanisms associated with fibrosis? Mass spectrometry based proteomics has been successfully used to study many diseases in order to identify potential biomarkers and/or understand pathogenesis, however, there haven't been many studies devoted to understanding proteins and their post-translational modifications (PTM) as connected with fibrosis in different tissues.

One of the reasons for this is probably technological - up to recent years the majority of proteomics techniques were not sensitive enough to identify and/or quantify minor changes in protein expression levels. The analysis of PTMs, on the other hand, is challenging because those modifications are usually in low abundance, complex or transient in nature. This overview will discuss mass spectrometry based proteomics techniques for protein characterization and point out those studies that have successfully used the approaches to study fibrosis and to search for potential biomarkers in fibrotic tissues or patients plasma.

## Discussion

Proteomics strives to characterize proteins and their role in complex biological systems. It is only one of the components of -omics approaches which - when fully applied to a biological problem - will generate a composite picture of biological processes. Mass spectrometry based proteomics is the first step to study the changes that proteins undergo as a consequence of fibrotic disorder, whether the changes are in protein levels (e.g. differential protein expression), temporal (following the development of a disorder), or on the post-translational level (e.g. phosphorylation) as compared to the control, healthy tissue or plasma. The final goal of many proteomics studies is the discovery of a biomarker protein or a protein panel that enables detection of disease, can monitor and measure the treatment progress and could also predict development of the disease. Majority of studies so far have used patient plasma to elucidate the mechanisms of fibrosis or find its putative biomarkers as compared to relatively few studies that use either tissues after biopsy or animal models/cell lines.

### Mass spectrometry based proteomics

Mass spectrometry is routinely used to identify proteins, quantify their levels (relative or absolute) and to characterize protein post-translational modifications. Some useful reviews on the application of different mass spectrometry based approaches were written by Yates et al. [1,2] and Cox et al. [3].

The advancements in the mass spectrometric technology, such as in instrument designs (dual pressure ion trap) and mass analyzers (orbitrap) have significantly improved the sensitivity and mass accuracy. Nevertheless, sample preparation and fractionation are very important especially when dealing with scarce clinical samples to be able to characterize high numbers of proteins and their post-translational modifications. Dealing with tissue samples or plasma poses additional challenges because of sample stability, solubility and abundance issues. Proteins in tissues are usually difficult to solubilize (which is critical for a successful tryptic digestion), whereas plasma contains abundant proteins (albumin) that can easily mask and thus prevent the identification of lower abundant proteins (such as signaling molecules and putative biomarkers). With the depletion of most abundant plasma proteins (up to 20), this approach then enables mass-spectrometry based proteomics to dig deeper into plasma proteome in order to identify low abundant proteins and their possible modifications.

The final goal of many proteomics experiments is to completely characterize a proteome: protein's identification, location, function, interacting partners, post-translational modifications (PTMs) and in many cases also the turnover rate of protein production/degradation or the rate of formation for a particular PTM. We would also like to know what are the occupancies for a particular PTM (e.g. what percentage of a given site in protein is phosphorylated). Some recent reviews on PTMs are the following: phosphorylation [4-6], acetylation [7], S-nitrosylation [8], glycosylation [9], ubiquitination [10], etc.

### Relative protein quantitation as a tool in biomarker discovery

**Figure 1:**
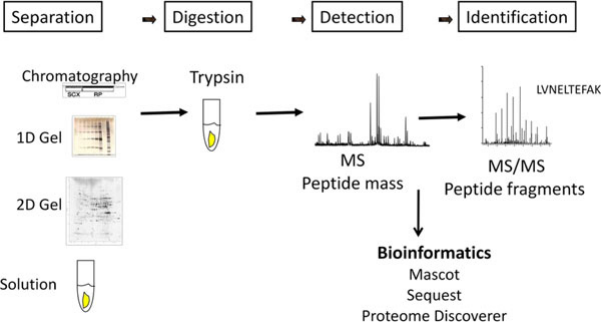
**Mass spectrometry based proteomics relies on the separation of proteins using gels or chromatography, digestion with an enzyme and further analysis by mass spectrometry**. Bioinformatics tools help in identification and quantification of proteins.

Mass spectrometry is an indispensible tool for relative protein quantitation which is usually applied to discovery of clinical biomarkers and mostly uses large-scale proteomics experiments. The first step in a typical large scale proteomics experiment is a separation technique on either the protein (e.g. 1D gel) or peptide level (e.g. SCX - strong cation exchange chromatography), followed by analysis of each of the individual fractions with liquid chromatography coupled to tandem mass spectrometry system (LC-MS/MS). The liquid chromatography is mostly reverse phase based with the flows in nanoliter range. Usually, 1D gel separation on protein level followed by an LC-MS/MS analysis gives a higher number of identified proteins because the first dimension separation is on the protein level as compared to SCX which separates peptides on the basis of charge. A summary of proteomics approaches using mass spectrometry is depicted in Figure 1.

The analysis of PTMs usually involves enrichment which is based on an antibody pull-down or it takes advantage of some physicochemical property of the PTM to selectively target proteins or peptides with that PTM. For example, tyrosine (Tyr) phosphorylated proteins (or peptides) are easily enriched by using a specific anti-pTyr antibody. On the other hand, serine or threonine phosphorylated proteins or peptides are enriched by using immobilized-metal affinity chromatography (IMAC) and metal-oxide affinity chromatography (e.g. TiO2 based). After the enrichment, the mass spectrometric analysis can pose some difficulties as well. A lot of PTMs are labile modifications that can easily fragment under collisionally induced dissociation in mass spectrometer [4].

There are several complementary approaches for relative protein quantification, such as DIGE (2D flourescence difference gel electrophoresis) [11], iTRAQ (isobaric tag for relative and absolute quantitation) [12] and SILAC (stable isotope labeling with amino acids in cell culture) [13], with DIGE being used the most in studies of fibrotic disorders.

Two-dimensional gels are an excellent way to separate proteins on the basis of their molecular weight and isoelectric point, enabling also visualization of some posttranslational modifications (such as phosphorylation). In the quantitative approach (DIGE), two protein samples are labeled with fluorescent dyes and when combined and separated on a 2D gel can be visualized by measuring the fluorescence showing whether a protein is up- or down regulated and for how much. The identification of individual spots of interest is carried out by mass spectrometry. The gels are usually scaled up (pick gel), stained with Coomassie blue, excised and digested with trypsin before being analyzed on a MALDI TOF/TOF or electrospray type of an instrument. Since fluorescence can be very sensitive, it is sometimes difficult to identify the proteins in spots by mass spectrometry which is a couple of orders less sensitive. Another drawback of this approach is that sometimes the individual spots may provide several identifications with similar proteins co-eluting.

As a complementary technique to DIGE, iTRAQ has recently gained a lot of popularity in the protein biomarker applications because of the ability to multiplex several samples in one run (up to 8) and because the number of identified proteins are usually higher.

### Proteomic analysis of fibrotic samples

Proteomic analysis of fibrotic tissue is further complicated with the limited ability of most common techniques to solubilize proteins in a complicated matrix. Usually, the number of identified proteins from a tissue sample is smaller as compared to a cell lysate. Most of the biologically important proteins (such as proteins involved in signaling mechanisms) are of lower abundance, thus it is essential to identify as many proteins as possible. The other drawback associated with fibrotic tissue analysis is limitation with the quantitation techniques because some of them due to the nature of labeling cannot be applied (e.g. SILAC vs. iTRAQ). Plasma samples, on the other hand, pose challenges as well, mainly due to high abundant proteins that are usually depleted to enable identification of less abundant proteins in the sample.

### Proteomics studies on organ fibrosis

#### Liver

Liver fibrosis has been relatively well studied using available proteomics tools and there are detailed reviews that critically discuss the use of proteomic approaches in hepatic fibrosis, including the search for putative biomarkers [14,15]. In a recent study, Lu et al. [16] carried out an extensive study using plasma samples from patients with and without chronic hepatitis B virus (HBV) infection. They used DIGE approach on pooled samples and identified potential biomarkers which were followed up by western blots on individual samples. Peroxiredoxin 2 is upregulated in fibrotic patients and could be potentially used in early diagnosis of HBV related liver fibrosis. Similar DIGE approaches were successfully used to study changes in the hepatic proteome after treatment with a traditional Chinese herbal medicine [17] and to discover novel biomarker candidate for liver cirrhosis [18-21]. Some of the approaches used tissue as starting material and others plasma with or without depletion of most abundant proteins.

#### Kidney

Renal fibrosis and complex proteomics associated with chronic kidney disease has been reviewed in a recent paper by Klein et al. [22]. Two other useful reviews focus on plasma and urine biomarkers of kidney disease [23,24]. A lot of discussion is devoted into pros and cons of using animal models (or even cell lines) to assess the markers for renal fibrosis. In a recent study, Dihazi et al. used a cell model with renal fibrosis phenotype coupled with DIGE to identify 30 regulated proteins in fibrotic compared to normal kidney fibroblasts [25].

#### Lungs

Idiopathic pulmonary fibrosis (IPF) - which is a disease of unknown etiology and is connected to progressive lung fibrosis - is the most studied condition related to lung fibroproliferative disorders. Kinulla et al. [26] reviewed the proteomics approaches applied to two different disease types: chronic obstructive pulmonary disease and idiopathic pulmonary fibrosis, pointing out that a lot of times proteomics studies compile the protein lists but fail to describe and elucidate the mechanisms behind these changes on the protein or PTM levels. Other useful reviews on lung-related fibrotic diseases and potential biomarkers were written by Lau et al. [27], Levine [28] and Govender et al. [29].

Among interesting proteins revealed and further studied by proteomics approaches, apolipoprotein A-I may be useful in therapeutic strategies after it was recently shown that its levels are decreased in IPT lungs compared to normal tissue animal models [30]. Similarly, another study revealed reduced levels of the receptor for advanced glycation end products (RAGE) protein which is a key player in cellular signaling [31].

#### Systemic sclerosis

Castro et al. [32] critically discussed biomarkers in systemic sclerosis, pointing out that the proteomics studies dealing with systemic sclerosis have mostly focused on pathophysiology rather than trying to find biomarkers connected with onset, progression and treatment of this condition. Another review by Abignano et al. [33] focuses on comprehensive -omics approaches to identify possible scleroderma biomarkers.

Scambi et al. [34] investigated sera from patients with scleroderma and patients with sclerodermatous graft-versus-host disease using DIGE proteomics approach to reveal 14 differentially expressed proteins, including factor H which could affect its binding to endothelial cells.

## Conclusions

Mass spectrometry based proteomics is a powerful tool to identify proteins and their posttranslational modifications. When applying these techniques to fibrotic disorders, the sample preparation is critical - with biological fluids it is possible to remove the most abundant proteins by immunodepletion. Liver fibrosis has been relatively well studied using available proteomics tools and there are several possible biomarkers that were identified by using proteomics approaches.

The current state of technology definitely brings along a lot of opportunities, and challenges as well, especially with the data analysis and making sense of the protein lists.

## List of abbreviations used

DIGE: 2-D Fluorescence Difference Gel Electrophoresis; PTM: post-translational modification; LC-MS/MS: liquid chromatography coupled to tandem mass spectrometry; iTRAQ: isobaric tag for relative and absolute quantitation; SILAC: stable isotope labeling with amino acids in cell culture.

## Competing interests

The author declares that they have no competing interests.
